# The Teaching of Hygiene in Board Schools

**Published:** 1891-06-06

**Authors:** 


### The Teaching of Hygiene in Board Schools.

Among the subjects taught in our elementary schools to girls it is satisfactory to find a place, modest but assured, allotted to "domestic economy." It is placed in the list of "specific subjects," but it would be difficult to say specifically what it includes. Domestic economy is, as every housekeeper knows, a wide subject when taken even in its narrowest sense, and in the meaning given to it by the education code it is by no means narrow. It includes the purchase of clothing and the prevention of infectious disease; the best methods of ventilation and the best joints for roasting, stewing, and boiling; the food values of meat and meal, and the remunerative values of investments; the getting up of fine linen and the constitution of the Post Office Savings Bank. Certainly a good deal of information, but somewhat miscellaneous in character; and when it has permeated the mind of the average school-girl or pupil-teacher it is apt to come out in somewhat eccentric form. Occasionally it is mere muddle, as when you are told that gratings should be put in the ceiling of a schoolroom to let the fresh air in, and near the floor to let the foul air out, but in many cases the answers, even when wrong, show a certain originality.

Take this example from an examination paper. The question was, "Why is lime-juice so much drunk on board ships?" One girl gave it as her opinion that sailors liked it because "they could not always get a proper dinner, and lime-juice was a change from tea or coffee." Others opined that lime-juice had been introduced to promote the cause of temperance, and indulged in moral remarks on the evils of drunkenness. The majority, however, knew of its use as an anti-scorbutic, but frequently added, "people on land get the same substance in the water they drink, which is more wholesome when it has percolated through limestone rock." Lime the fruit and lime the stone were clearly one to them.

Questions on water supply bring out varied notions on the subject. "Why is absolutely pure water not the best for drinking?" was asked recently. By "pure" water the examiner meant chemically pure—$H_2O$, only that and nothing more—but a good many of the examined took up the word in its popular sense. One of these boldly said that "the proposition that pure water was not the best might reasonably be disputed," which, from her point of view, and that of all of us who are not given to speaking technically, was true enough; but no other had the courage to quarrel with the mysterious powers whose function is to "set papers" for the bewilderment of the average mind, and many replied with meek illogicality that "pure water is not the best for drinking because of its impurities." Then they waxed eloquent on insects caught and drowned by falling raindrops, and mysterious "beasts" which the microscope revealed. Some even dwelt on the contaminating influence of sewage, apparently without a suspicion that water so tainted could not be regarded from any point of view as "pure."

It will be remembered that the schoolmaster in one of Bret Harte's stories fell in love with Mliss, the daring pupil who, not finding in her astronomy book any allusion to Joshua's making the sun and moon stand still, emphatically expressed her disbelief in the miracle ever having taken place. One can sympathise with that schoolmaster when, having dealt with a score of answers from people who "never think of thinking for themselves at all," one meets with the answer of one who, rightly or wrongly, does. "When is fish a desirable article of food?" is asked. A hundred tell you that, among other uses, it stimulates the brain; only one student at a training college has the courage to add, "but having been a teacher in a fishing village I am inclined to doubt the value of fish in this matter." Evidently the lads and lasses she had taught there had shown but little of the mental activity which should have resulted from a fish diet.

A comment like this is a better testimony to the intelligence of the student than many an answer that is, after a fashion, correct. The statement that "tapioca is derived from a poisonous root, and is very good for invalids and young children" is true enough, but the fashion of putting it is not happy. It is not calculated to make invalids and young children relish their tapioca. One girl, whose acquaintance with oxalic acid was evidently theoretic—and phonetic!—gave it as her opinion that ink stains are best removed by "salts of sorrow"—the phrase suggests tear-drops, which have often enough been caused by spilled ink. Another dubs the same bleaching agent "salts of coral." Still it is on points like these that the students show to best advantage. They may not be able to give any clear account of the physiological action of food, but in general they know how to cook it; they have some practical notion of woman's mission in the world. Sometimes, indeed, their ideas are extreme and suggest descent from countless generations of mothers who had no idea of their "rights." One, showing how three children and their parents may dine for a shilling, advises that sixpence be spent on bones and vegetables to make soup for the youngsters and their mother, "which will leave sixpence for a bit of meat for father." Another of the same type, speaking of exercise, gives it as her opinion that "housework is the best form of exercise for the female." This opinion will very probably be endorsed by "the male" or at least by a majority of him; but, sad to say, there are women to whom even unlimited cooking and carpet-sweeping are not the supremest joys.

When questions as to clothing are asked, the feminine instinct comes out. Not avowedly; one would think that a marked antagonism to anything frivolous or decorative was a distinguishing mark of the sex. "No sensible person would think of following the fashions now-a-days; they are too ridiculous," says one, who perhaps would like to go back to the ruffs and farthingales and other rational habiliments of Queen Elizabeth's time. "Last summer's hats consisted of only a spray of flowers and a bit of ribbon," is the scornful cry of another. They all—if one may believe them—desire to dress in blue serge and grey homespun; they scorn flowers and feathers, and crave only for the simplest hats, shady in summer, and in winter "of a sensible shape, like a pork-pie," but how they enlarge on the subject! They are hazy about the kinds of clothing that are bad conductors of heat, and incline to number among these "linen, because it feels cold when you put it on," but about these fashionable gowns and bonnets which they despise so greatly their opinions are clear, decided, and full. "Methinks the lady doth protest too much."

On these points they moralise not a little. "Oh! how careful we should be not to waste in vanity money that might help others, or be invested for old age," writes one; and everyone will feel how natural such a sentiment is in a girl of eighteen. Sometimes the moral tone so outweighs the sense of humour that the narrow bound between the sublime and the ridiculous is overleaped. "Water, that great gift of Providence, is usually brought into town in pipes, and stoneware ones are better than lead." Most true and practical, if somewhat infelicitous in expression! "Without food life could not long be sustained at a temperature of 98° Fahr." is a sentence that gives us almost a superfluity of information. This determination to crowd into an answer all they know, and, indeed, sometimes more, is characteristic of the majority of the candidates for examination. It is an amiable weakness, and, after all, an inability to express ideas with clearness and proportion by no means implies an inability to apply them practically. Domestic economy, as taught in the schools, may give some strange results in examination papers, but none the less does it give a sound training in a subject of which no woman should be wholly ignorant—above all, women of the class to which these girls belong. We yield to none in respect for the *femmes savantes* of our day and the work they have done, but yet, like Chrysale in Molière's play, when it comes to the accomplishments of the lady who presides in the kitchen, we are more concerned that she should make good soup than know how to turn a sonnet.

